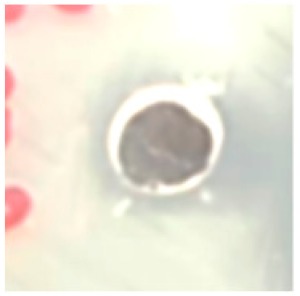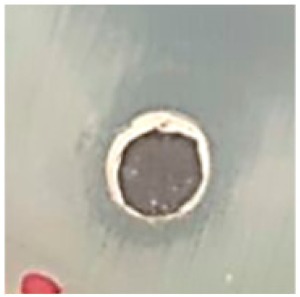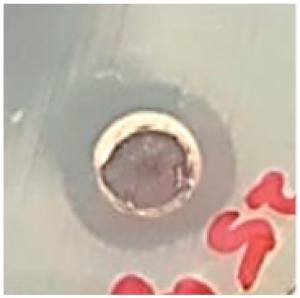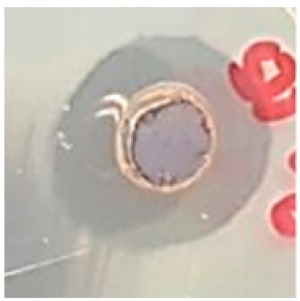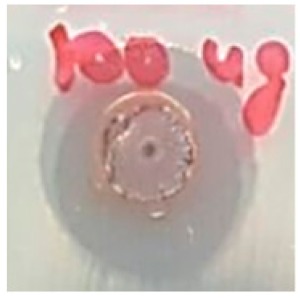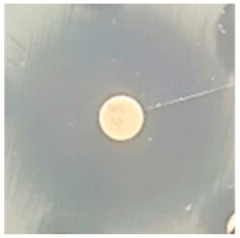# One-step Synthesis of Silver Nanoparticles Using Saudi Arabian Desert Seasonal Plant *Sisymbrium irio* and Antibacterial Activity Against Multidrug-Resistant Bacterial Strains

**DOI:** 10.3390/biom9110662

**Published:** 2019-10-28

**Authors:** Suresh Mickymaray

**Affiliations:** Department of Biology, College of Science, Al-Zulfi-, Majmaah University, Majmaah 11952, Riyadh Region, Saudi Arabia; s.maray@mu.edu.sa or drsuresh.maray@gmail.com; Tel.: +966-582-315-293

**Keywords:** *Sisymbrium irio*, silver nanoparticles, characterization, ventilator-associated MDR pathogens, antibacterial activity

## Abstract

Globally, antimicrobial resistance has grown at an alarming rate. To combat the multidrug-resistant (MDR) superbugs, silver nanoparticles (Ag NPs) were synthesized using an aqueous leaf extract of seasonal desert plant *Sisymbrium irio* obtained from the central region of Saudi Arabia by a simple one-step procedure. The physical and chemical properties of the Ag NPs were investigated through ultraviolet visisble analysis (UV-vis), Fourier-transform infrared (FTIR) spectroscopy, X-ray diffraction (XRD), scanning electron microscope (SEM), and transmission electron microscope (TEM) analysis. The UV-vis spectrum showed an absorption band at 426 nm. The XRD results showed a highly crystalline face-centered cubic structure. The surface morphology analyzed using SEM and TEM analyses showed the particle size to be in the range 24 nm to 50 nm. Various concentrations of Ag NPs were tested against MDR *Pseudomonas aeruginosa* and *Acinetobacter baumanii* that cause ventilator-associated pneumonia (VAP). American Type Culture Collection (ATCC) *Escherichia coli*-25922 was used as the reference control strain. The Ag NPs effectively inhibited tested pathogens, even at the lowest concentration (6.25 µg) used. The bacterial inhibitory zone ranged from 11–21 mm. In conclusion, the newly synthesized Ag NPs could be a potential alternative candidate in biomedical applications in controlling the spread of MDR pathogens.

## 1. Introduction

For the past two decades, the interest in the synthesis of ecofriendly metal and metal oxide nanoparticles has grown dramatically in the research fields of material science and engineering and biotechnology. Because of the high ‘surface-to-volume ratio’ of the nanoparticles, they have unique properties of size, shape, self-assembly, and conductivity. Among the widely studied silver (Ag) [1], gold (Au) [2], platinum (Pt) [3], and palladium (Pd) [4] nanoparticles, Ag NPs have gained much attention due to their various applications in gas sensors [5], dye-sensitized solar cells [6], textiles [7], water purification systems [8], cosmetics [9], and food packaging [10]. They show antimicrobial [11], larvicidal [12], anti-cancer, dye degrading [13], and antibiofilm forming [1] properties. Conventional methods of synthesis of nanoparticles are high cost, utilize more energy, and consume more time. The chemical reducing agents used cause environmental issues. Hence, these issues must be overcome, and actions are needed to determine an alternate method for the synthesis of nanoparticles. Generally, methods of green synthesis of nanoparticles use algae, fungi, and various lower and higher plants [12]. Plant-based synthesis is more convenient than microbial method since the latter suffers from the high cost of culture media, time consumption for growth enhancement, difficulty in handling the process, and requirement of biological knowledge. Usually, different plant parts, such as the leaf, bark, root, stem, seed, flower, fruit, and tubers, have been used for nanoparticle synthesis [14]. The plant materials are selected based on the content of bioactive phytoconstituents in the exact plant part through the knowledge gained from studying the literature. The leaf part of plants is preferred by many researchers for the synthesis of metal nanoparticles due to the rich content of bioactive phytoconstituents in leaves, their easy availability, and readiness for instant use [15]. The bioactive phytoconstituents act as capping, as well as reducing agents, for the synthesis of nanoparticles. Previously, several attempts have been made for the synthesis of Ag NPs using leaf extracts of plants like *Dodonaea viscosa* [11], *Gloriosa superba* [16], *Lilium lancifolium* [1], *Salvia spinosa* [17], *Mukia scabrella* [18], and *Syzygium alternifolium* [19].

*Sisymbrium irio* L. belongs to the family of Cruciferae. It is widely distributed in the deserts of the central region of Saudi Arabia during the winter season between January and May. It is used for dietary purposes due to its high nutrient and protein (35%) content. This plant contains several phytoconstituents like glycosides, triterpenes, amino acids, coumarin, tannins, flavonoids, alkaloids, and saponins [20,21]. The ethanol extract of seeds has shown better antibacterial, antipyretic, and analgesic activities, while the n-hexane extract of aerial parts exhibit better antibacterial and cytotoxic activities.

At present, the antimicrobial resistance-related infectious diseases, including hospital-acquired Gram-negative bacterial infection and its associated morbidity and mortality, have grown at an alarming rate. Most of the presently available antibitics have become ineffective to treat and control the multidrug resistant bacterial pathogens globally [22,23,24,25].

The multidrug-resistant bacteria, mainly *Pseudomonas aeruginosa* and *Acinetobacter baumannii*, are the most important nosocomial pathogens in the healthcare-associated environment and cause high mortality [26,27,28,29]. The infections caused by these two species are difficult to treat because they are naturally resistant to many antibiotics and develop new resistance mechanisms continuously. Furthermore, these two bacteria can be transmitted through personal contact (direct) or contaminated environments (indirect contact) [30]. Moreover, *A. baumannii* can easily spread in hospital environments and survive for long periods [31]. For the reason, the nosocomial infections by these two pathogens have become a major health concern in many hospitals worldwide [32].

Typically, medicinal plant extracts have been used for their antibacterial potential, which is due to the presence of bioactive principles. However, the antibacterial activity of plant bioactive has some limitations, such as dependency on dosage concentration, applicability on a narrow range of bacterial species, and low stability [33]. Recently, Lim et al. reported that actinomycetes isolated from soil showed better antibacterial activity against multidrug resistant bacteria [34]. However, these methods are difficult to follow, and consume more time for growing the media. Keeping the above issues in mind, the present study explored Ag NPs synthesized by a green method for the effective growth inhibition of the multidrug resistant bacteria with low concentration.

This study demonstrated a simple methodology for the synthesis of Ag NPs using the leaves extract of *S. irio* available in the central region of Saudi Arabian deserts during the winter. The advantages of this study are: (i) Room temperature process, (ii) less time consumption (15 min), (iii) reliability, (iv) simplicity, and (v) mass production. As per the existing literature and knowledge, this is probably the first report on the synthesis of Ag NPs using *S. irio* leaves extract. The Ag NPs were characterized and the crystal structure, optical and morphological properties were investigated. The effect of different concentrations of Ag NPs was investigated against nosocomial multidrug-resistant Gram-negative bacterial strains *P. aeruginosa* and *A. baumannii* with American Type Culture Collection (ATCC) *Escherichia coli*-25922 strain as the reference. This finding may help in the field of medicinal research to control the multidrug resistant-related morbidity and mortality.

## 2. Materials and Methods 

### 2.1. Chemicals 

Silver nitrate (AgNO_3_), Mueller Hinton Agar (MHA), and Nutrient Broth (NB) were procured from HiMedia Laboratories Pvt. Ltd (HiMedia, Mumbai, India). For all the experiments, analytical grade reagents and ultrapure water (Milli-Q) were used.

### 2.2. Collection of the Plant Material 

**Figure 1:**
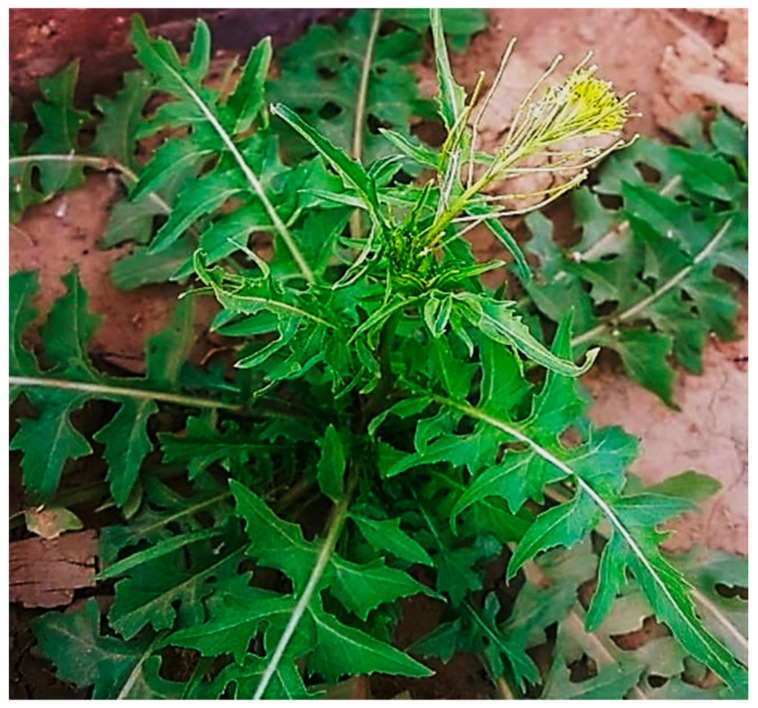
Saudi Arabian desert seasonal plant of *Sisymbrium irio.*

The plant leaves were collected during the winter season (Location: 26.3158330N; 44.8608130E) at the College of Science, Al Zulfi campus, Kingdom of Saudi Arabia (Figure 1). The deposited voucher specimen bearing the no: 22185 can be found in the herbarium in the Botany Department of King Saud University, Riyadh, Kingdom of Saudi Arabia.

### 2.3. Collection of Bacterial Pathogens

The ventilator-associated pneumonia-causing multidrug-resistant *P. aeruginosa* and *A*. *baumannii* and the reference strain ATCC-25922 *E. coli* were obtained from the culture stock maintenance at Microbiology Laboratory, College of Science, Al Zulfi, Kingdom of Saudi Arabia.

### 2.4. Identification of Bacterial Strains

The bacteria were identified and their antimicrobial drug resistance was detected by VITEK 2 compact automated system using a GN: 21341 identification card and AST-N 292 antimicrobial susceptibility cards, respectively (BioMérieux, Durham, NC, USA). The bacterial pathogens resistant to more than one antibiotic were considered as multidrug-resistant. The susceptibility pattern was interpreted as per the recommendations of European Committee on Antimicrobial Susceptibility Testing (EUCAST) as shown in Table 1.

### 2.5. Synthesis of Ag NPs

For the preparation of aqueous leaf extract of *S. irio*, fresh leaves were collected and washed in running tap water and rinsed well with the Ultrapure Milli Q water. Then, 10 g of leaves were chopped into 1–2 cm. Cut leaf pieces were inoculated into 100 mL of Ultrapure Milli Q water in a 250-mL conical flask and boiled for 10 min. Following this, the aqueous leaf extract was filtered through Grade No.1 Whatman filter paper (125 mm). The aqueous leaf extract was collected in a conical flask and stored at room temperature (25 ± 2 °C) for further use.

About 50 mL of *S. irio* aqueous leaf extract was added to 450 mL of 1mM AgNO_3_ solution and stirred at 35 °C using a magnetic stirrer. After 15 min, a yellowish-brown color was observed, indicating the formation of Ag NPs [35]. The solution containing the Ag NPs was transferred into a watch glass and incubated at 60 °C for 24 h. The solution containing Ag NPs/dry powder was used for structural, morphological, and optical characterization, as well as for assessing the antibacterial activity.

### 2.6. Characterization

The Ag NPs were subjected to UV-visible analysis (UV-vis, 200 nm to 800 nm) using a Shimadzu spectrophotometer (Model UV-1800, Kyoto, Japan) operating at a resolution of 1 nm. Fourier-transform infrared spectroscopic (FTIR) analysis of the nanoparticles was done using a Spectrum-65 FTIR spectrometer (PerkinElmer Co., Ltd., Waltham, MA, USA). The FTIR measurement of the sample was taken in the wavenumber region 4000 cm^−1^ to 400 cm^−1^ using 16 accumulated scans in the attenuated total reflection mode with diamond/ZnSe (one reflection) crystal. The structural analysis of Ag NPs was carried out using a powder X-ray diffractometer (PANalytical X’Pert Pro, PANalytical X’Pert Pro, Carnation, WA, USA) with Cu-Kα radiation (wavelength:1.5418 Å) with nickel monochromator in the range of 2θ from 10° to 80°. The nano-crystallite domain size was calculated from the width of the XRD peaks using Debye-Scherrer’s formula D = 0.9λ/βcosθ [36]. The surface topology of Ag NPs was studied by scanning electron microscope (SEM, EVO 18 SEM, Carl Zeiss, Jena, Germany). For accuracy, morphological structures and actual particle size were analyzed under transmission electron microscope (TEM, JEOL Electron Optics Laboratory, Tokyo, Japan) operated at 200 kV. To facilitate TEM analysis, a drop of solution containing the Ag NPs was dispensed to a carbon-coated 200 mesh copper grid and allowed to airdry at room temperature just before the examination.

### 2.7. Antibacterial Assay

The antibacterial activity of the synthesized Ag NPs was tested against *P. aeruginosa, A. baumannii* with the reference strain ATCC *E. coli*-25922 by agar well diffusion method [37]. The overnight bacterial cultures were standardized using the McFarland standard (10^6^ cfu/mL). The silver nanoparticle suspension was prepared by mixing 1 mg of Ag NPs in 1 mL of distilled water (1 mg/1 mL concentration). The nanoparticle suspension was sonicated for 15 min for dispersion of the particles. Mueller–Hinton plates were prepared and each bacterial strain was spread uniformly onto the plates using spread plate technique aseptically. After spreading the bacteria, five wells of 6 mm diameter were made in each plate using a sterile cork borer. Using sterile micropipette tips, each culture plate was added with five different concentrations (6.25, 12.5, 25, 50, and 100 µg) of the nanoparticle suspension. A standard antibiotic disc Meropenem (10 µg) was used as a positive control. The culture plates were incubated at 37 °C for 24 h. After incubation, antibacterial activity was determined by measuring the zone of inhibition from each well and the experiment was conducted in triplicates.

### 2.8. Statistical Analysis

All the results were reported as mean ± standard error of three independent biological replicates. We employed ANOVA followed by Tukey’s HSD test (*p* ≤ 0.05) to analyze the data from the antibacterial activity with the help of the SPSS software package 16.0 version.

## 3. Results

### 3.1. UV-Vis Spectroscopic Analysis 

**Figure 2:**
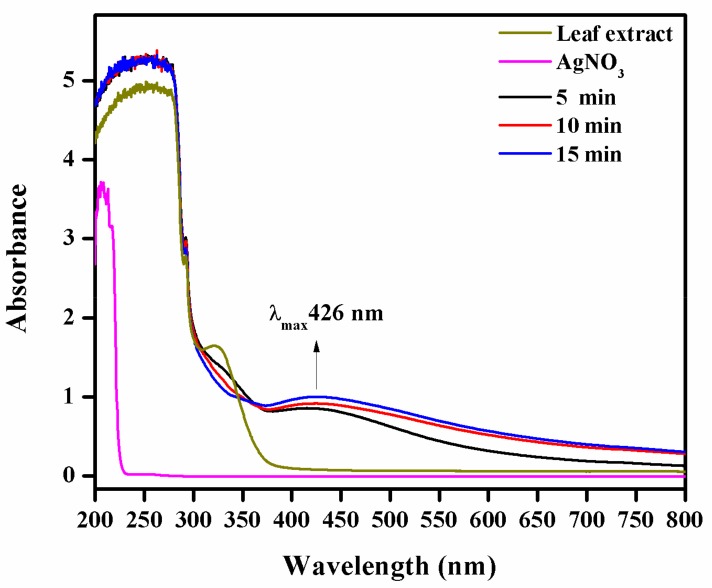
Ultraviolet visible (UV-vis) analysis of Ag nanoparticles (NPs).

The *S. irio* leaf extract showed an absorption peak at 326 nm due to the π → π* transition taking place in the plant bioactive compounds (Figure 2). The Ag NO_3_ solution did not show any absorption in the UV-vis spectrum. The reaction mixture containing AgNO_3_ solution and the leaf extract monitored at 5, 10, and 15-min time intervals exhibited absorbance peak at 426 nm [16].

### 3.2. FTIR Analysis of Synthesized Ag NPs 

**Figure 3:**
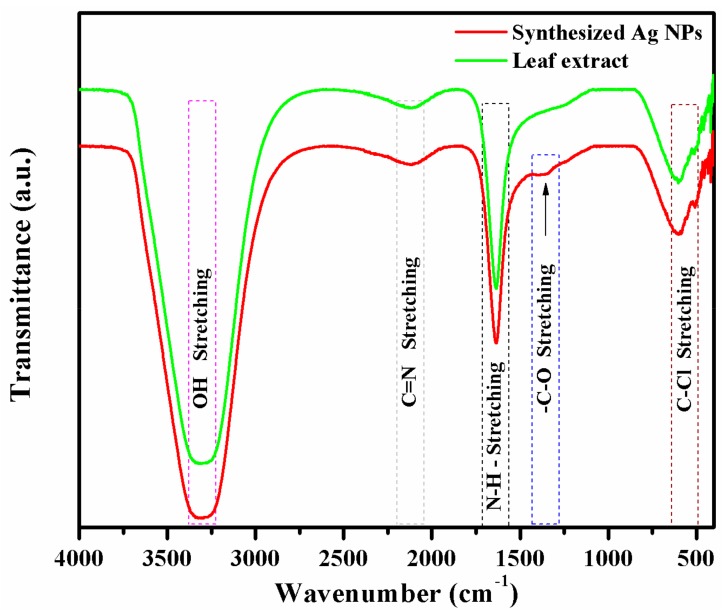
Fourier-transform infrared (FTIR) spectrum of leaf extract and synthesized Ag NPs.

FTIR spectroscopy is commonly used to identify the organic and inorganic functional groups of material. The FTIR spectra of *S. irio* leaf extract and the synthesized Ag NPs are shown in Figure 3. Both the spectra showed bands at 3309, 2117, 1630, 602, and 508 cm^−1^. The strong band at 3309 cm^−1^ corresponds to alcoholic O-H stretching. The weak band at 2117 cm^−1^ is owing to the C=N stretching of R–N=C=S. The sharp band at 1630 cm^−1^ can be assigned to N–H stretching. The lower frequency bands at 602 and 508 cm^−1^ can be assigned to N-H bending C-Cl stretching vibrations. In the IR spectrum of the Ag NPs, a new band, observed at 1337 cm^−1^, corresponds to the –C–O stretching [38,39,40].

### 3.3. XRD Analysis

**Figure 4:**
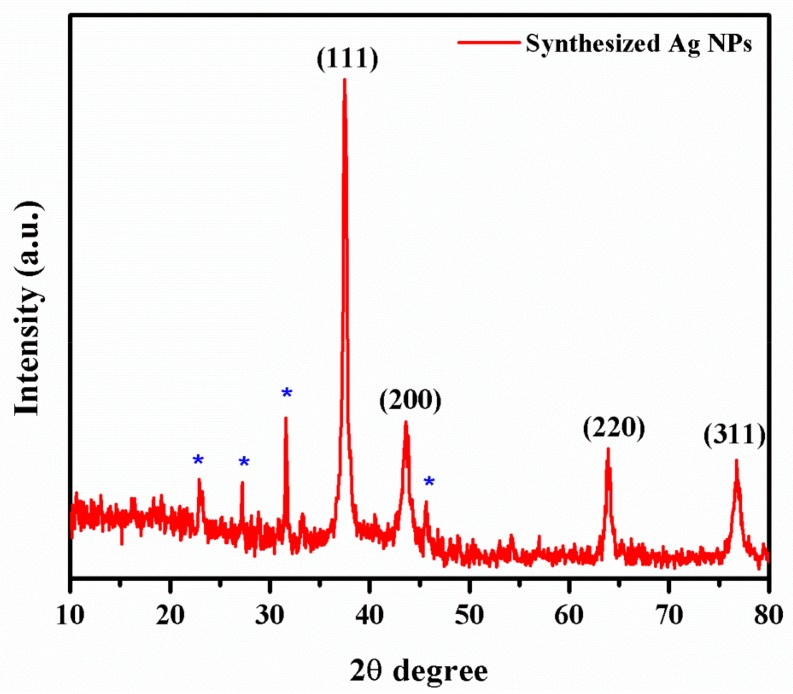
XRD analysis of Ag NPs.

The powder X-ray diffraction patterns revealed the nanocrystalline nature of Ag NPs as shown in Figure 4. The XRD patterns indicated four intense peaks at angles (2θ) 38.11, 44.27, 64.22, and 77.47°, corresponding to (111), (200), (220), and (311) planes, respectively [41].

### 3.4. SEM, TEM, and Selected Area Electron Diffraction (SAED) Analysis of Ag NPs 

**Figure 5:**
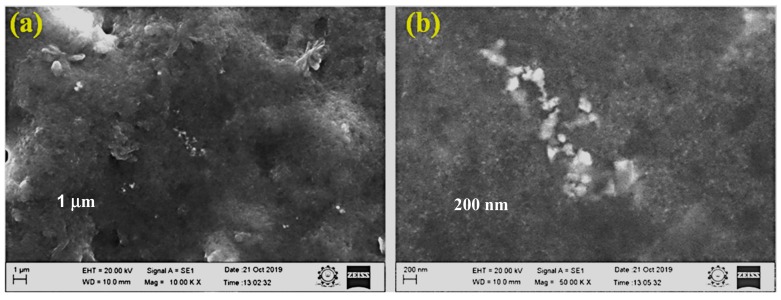
(**a**,**b**) SEM image of Ag NPs.

**Figure 6:**
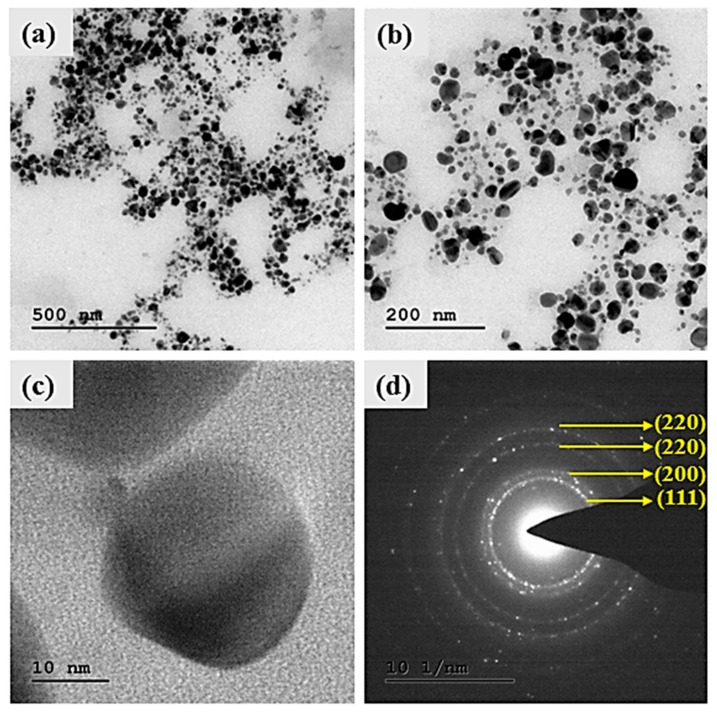
TEM image: (**a**) 500 nm, (**b**) 200 nm, (**c**) 10 nm, and (**d**) selected area diffraction electron (SAED) patterns of Ag NPs synthesized from *S. irio.*

The surface morphology of the synthesized Ag NPs was studied by SEM analysis (Figure 5a,b). The SEM images illustrated agglomerated spherical-shaped nanoparticles with sizes in the range of 50 nm [42]. The crystallographic structure and accurate particle size of the Ag NPs were determined by the TEM analysis. The Ag NPs showed spherical morphology with sizes ranging from 35 to 50 nm, as shown in Figure 6a–c [42].

### 3.5. Antibacterial Activity

**Figure 7:**
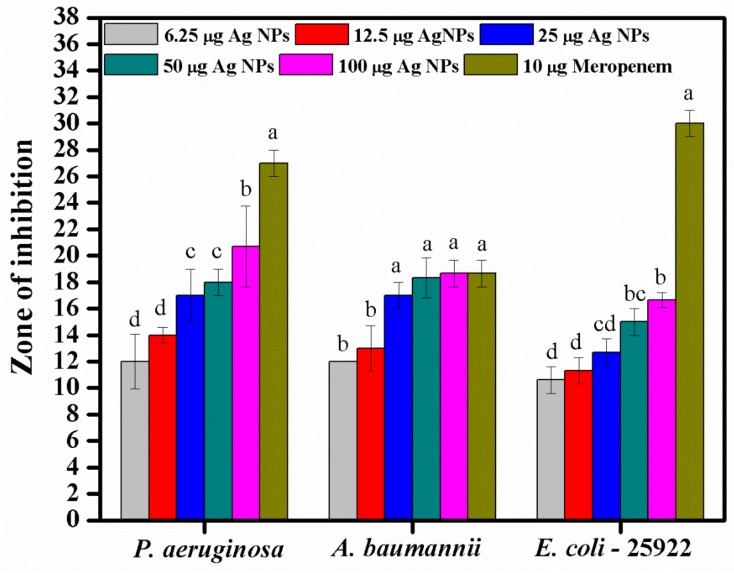
Antibacterial activities of the synthesized Ag NPs and the standard antibiotic Meropeneom (10 µg) against ventilator-associated pneumoniae causing multidrug-resistant gram-negative clinical pathogens with the reference strain ATCC *E. coli*-25922. The T-bars represent standard errors. The different small alphabet letters above each column indicate significant differences (ANOVA, Tukey’s HSD test, *p* ≤ 0.05).

The antibiotic-resistant characteristics of *P. aeruginosa* and *A. baumannii*, which cause ventilator-associated pneumonia, and the reference strain ATCC *E. coli*-25922 are shown in Table 1. From the results, it was observed that *P. aeruginosa* and *A. baumannii* exhibit similar resistance characteristics against the tested antibiotics (resistant to nine antibiotics and susceptible to four antibiotics). The reference strain ATCC *E. coli*-25922 was susceptible to the entire list of antibiotics as expected. In order to evaluate the antibacterial activity, different concentrations (6.25, 12.5, 25, 50, and 100 μg) of the synthesized Ag NPs were tested against MDR *P. aeruginosa, A. baumannii,* and ATCC *E. coli*-25922 by the well diffusion method (Table 2). All the tested concentrations exhibited the zone of inhibition and the antibacterial activity increased with an increase in the concentration of Ag NPs, which proves a dose-dependent response (Figure 7). Among them, the 100-μg concentration showed excellent antibacterial activity on *P. aeruginosa,* followed by *A. baumannii* and ATCC *E. coli*-25922.

## 4. Discussion

In the synthesis of Ag NPs using the selected seasonal desert plant leaves, a slow visual color change from colorless to brown was observed. This color change confirmed the reduction of Ag^+^ into Ag^0^. The UV-vis spectrum of the mixture at this stage showed a strong absorption peak at 426 nm corresponding to the wavelength of the surface plasmon resonance (SPR) of Ag NPs. SPR is a collective oscillation of conduction band electrons in metal nanoparticles under the influence of excited electromagnetic incident light. The SPR band is used to determine the nanoparticle size and geometry. A blue shift indicates a decrease in the particle size, while a redshift reveals an increase in the particle size. The rapid reduction process depends on the content of bioactive phytoconstituents, pHn and temperature. This trend is well-consistent with earlier research [43,44,45,46,47].

In FTIR spectrum of *S. irio* leaf extract, the bands observed at 602 cm^−1^ and 508 cm^−1^ were due to the bending vibrations of N–H groups in proteins and C–Cl stretching, respectively. The functional groups indicated the presence of primary bioactive phytocompounds, such as glycosides, triterpenes, amino acids, coumarin, tannins, flavonoids, alkaloids, and saponins [20,21], in the *S. irio* leaf extract. Remarkably, a new band was observed at 1337 cm^−1^ in the spectrum of the synthesized Ag NPs corresponding to –C–O stretching mode, which indicated the reduction of Ag^+^ ions to Ag^0^ NPs [38,39,40]. In turn, the transparency of synthesized Ag NPs decreased to a small extent when compared the leaf extract, which also indicated the reduction of the metal ions to metal nanoparticles. Our results showed good correlation with a previous report [1].

In the powder XRD pattern, there were four intense peaks at angles (2θ) 38.11, 44.27, 64.22, and 77.47°, corresponding to (111), (200), (220), and (311) planes, respectively. All reflections can be indexed to the face-centered cubic nature of synthesized Ag NPs. The characteristic peak values were confirmed by the Joint Committee on Powder Diffraction Standards (JCPDS File No: 04-0783). Some of the unassigned peaks observed in the XRD pattern were related to the phytocompounds present on the surface of Ag NPs. From the XRD data, the calculated average crystallite size was 21.59 nm. A similar observation has been made in recent studies [48,49,50].

The results of the SEM analysis showed the surface morphology of the synthesized Ag NPs to be agglomerated spherical-shaped with sizes ranging up to 50 nm. The TEM analysis revealed the spherical appearance of Ag NPs with sizes ranging from 35 nm to 50 nm. The particles were uniformly distributed without any aggregation. These results correlated and were in well-agreement with previous studies [43,51,52]. The selected area electron diffraction (SAED) pattern showed well-defined, spotty rings, implying the polycrystalline nature of the nanoparticles, which well-matched with the XRD results, as shown in Figure 6d. The d-spacing values were calculated using the following equation [53]:

Lλ = dR(1)

According to the literature, antibiotic development continues to stagnate. There has been no new class of antibiotics discovered since 1987 for the treatment of MDR pathogens inclusive of systemic gram-negative bacterial infections. Further, the World Health Organization (WHO) published a list of antibiotic-resistant bacteria as “Priority pathogens”, which need new antibiotics urgently [54]. The Ag NPs synthesized in this study using *S. irio* leaves proved potential antibacterial efficacy against the selected intrinsic MDR pathogens. The antimicrobial-resistant characteristics of *P. aeruginosa* and *A. baumannii* was assessed by the VITEK-2 automated system. On the other hand, the reference strain ATCC *E. coli*-25922 was susceptible to the entire list of antibiotics as expected. A similar trend was reported by Nys et al. (2018) [55,56]. The inhibitory zones of *P. aeruginosa* were in accordance with the results reported by Singh et al. (2014) [40]. The authors synthesized Ag NPs from *Tinospora cordifolia*, which showed zones of inhibition ranging from 11 mm to 18 mm at the concentration of 100 µg/mL against 20 MDR *P. aeruginosa* strains. Green synthesized Ag NPs have been shown to be powerful antibacterial agents against MDR *P. aeruginosa* and *A. baumannii* by several authors [57,58,59]. The size and concentration of the nanoparticles play a key role in the antibacterial activity.

The exact mechanism of antibacterial activity of the Ag NPs remains unclear. However, acceptable mechanisms have been proposed in the literature as follows: Ag NPs come into contact with the negatively charged bacterial cell membrane by an electrostatic interaction, leading to the disorganization of the membrane permeability and leakage of bacterial electrolyte, thus causing damages to the structure and function of mesosomes. The silver nanoparticles react with sulfhydryl (-SH) groups inside the cytosol, inactivate the protein synthesis, and suppress the activity of the enzyme. The interference with the intracellular cell signaling reduces the ATP synthesis, which increases the ROS generation and causes cell death [1,16,20].

## 5. Conclusions

A simple and rapid method for the synthesis of Ag NPs using leaf extract *S. irio* at room temperature (27 °C) without any chemicals was devised. The synthesized Ag NPs were thoroughly studied for their physicochemical properties. The antibacterial activity of the nanoparticles against MDR nosocomial pathogens causing ventilator-associated pneumonia (VAP) was investigated in detail. The Ag NPs potentially inhibited the selected pathogens even at the lowest concentration. The UV-vis spectrum revealed an absorbance band at 426 nm that confirmed the reduction of Ag^+^ metal ions to Ag^0^ NPs. In the FTIR spectrum of the Ag NPs, a new vibration band at 1337 cm^−1^ corresponding to –C–O stretching appeared due to the reduction influenze of glycosides and tannins. At the same time, the transparency of Ag NPs decreased when compared to that of the pure leaf extract. The powder XRD studies showed that the Ag NPs had a face-centered cubic structure with good crystalline nature. The surface morphology of the synthesized Ag NPs exhibited a spherical shape with sizes ranging from 35 to 50 nm. The antibacterial activity of the Ag NPs showed a dose-dependent response. The lowest concentration used in this study, 6.25 µg, effectively inhibited the intrinsic MDR pathogens *P. aeruginosa* and *A. baumannii*. In accordance with the present situation, there is an urgent need to decrease the burden of existing antibiotics and slow down the development of antibiotic resistance through an alternative method. Therefore, the synthesized Ag NPs gained the attention of many researchers. Silver nanoparticles may find applications in various hospital-associated surgical accessories, catheters, wound-healing fabric dressings, antimicrobial papers, and as an additive in pharmaceutical products to reduce and manage the MDR bacterial infections and spread.

## Figures and Tables

**Table 1:** The antimicrobial-resistant pattern of selected multidrug-resistant pathogens.

Antibiotics Group	Concentration (µg)	*P. aeruginosa*	*A. baumannii*	*E. coli* (ATCC: 25922)
**Aminoglycosides**				
Amikacin (AN)	8; 16; 64	R*	R	S**
Gentamicin (GM)	4; 16; 32	R	R	S
Tobramycin (TM)	8; 16; 64	R	R	S
**Carbapenems**				
Ertapenem (ETP)	0.1; 6	S	S	S
Imipenem (IPM)	1; 2; 6; 12	S	S	S
Meropenem (MEM)	0.5,2,6,12	S	S	S
**Cephalosporins**				
Ceftazidime (CAZ)	1; 2; 8; 32	R	R	S
Cefepime (FEP)	2; 8; 26; 32	R	R	S
**Fluoroquinolones**				
Ciprofloxacin (CIP)	0.5; 2; 4	R	R	S
Levofloxacin (LEV)	0.25; 0.5; 2; 8	R	R	S
**Tetracyclines** Tigecycline	0.75; 2; 4	R	R	S
**Polypeptides** Colistin (CS)	4; 16; 32	S	S	S
**Co-Trimoxzalole** Trimethoprim/Sulphonamides	1/19; 4/76; 16/304	R	R	S

R*: Resistant; S**: Susceptible. Organisms and their multidrug-resistant characteristics were studied using the GN: 21341 card and AST-N 292 card of the VITEK 2 automated system, respectively. (BioMérieux, Durham, USA). The antimicrobial susceptibility pattern was interpreted as per the European Committee on Antimicrobial Susceptibility Testing (EUCAST) recommendations (http://www.eucast.org/clinicalbreakpoints).

**Table 2:** Antibacterial activities of the synthesized Ag NPs against *P. aeruginosa*, *A. baumannii,* and *E. coli.*

Organism	Concentration of Ag NPS					Meropenem
	6.25 µg	12.5 µg	25 µg	50 µg	100 µg	(10 µg)
	Antibacterial Effect Against VAP Causing MDR Pathogens and Reference Strains					
*P. aeruginosa*	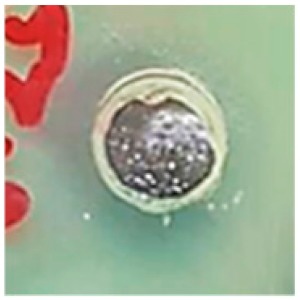	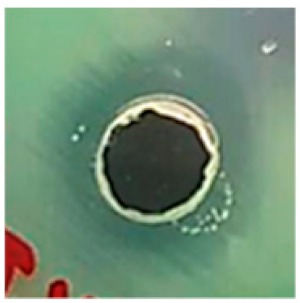	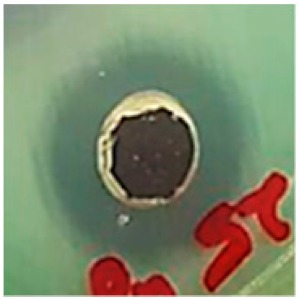	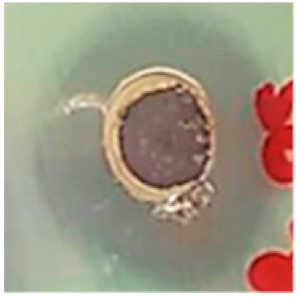	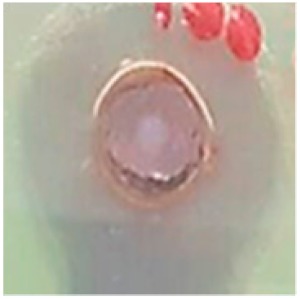	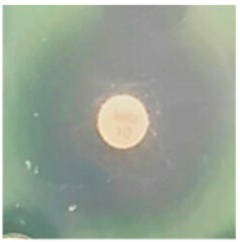
*A. baumannii*	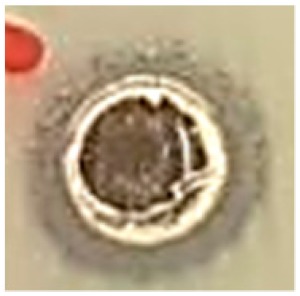	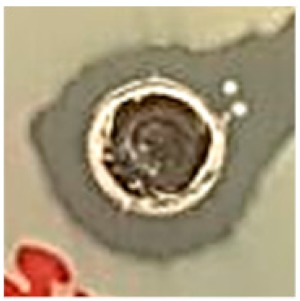	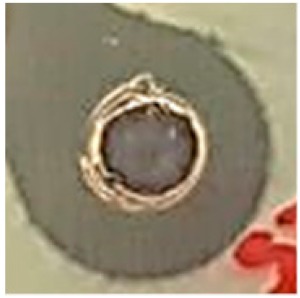	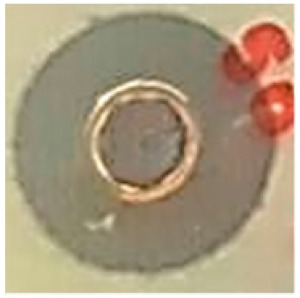	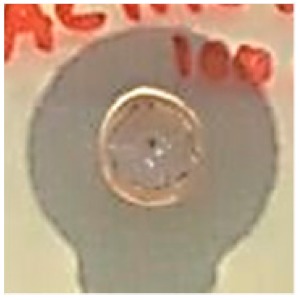	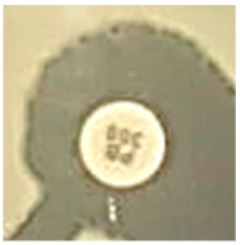
*E. coli* (ATCC-25922)